# Supplementary material for: Resolving the full spectrum of human genome variation using Linked-Reads
Source: Genome Res. 2019 Apr;29(4):635–45. doi: 10.1101/gr.234443.118 (PMC6442396; doi:10.1101/gr.234443.118)
Supplement: Supplemental Material [file supp_gr.234443.118_Supplemental_longranger-2.2.zip › longranger-2.2.tar.bz2!/lr2.2-public/lib/stan/cmdstan/stan/doc/api/html/agrad_2fwd_2matrix_2qr___r_8hpp_source.html]

Stan: src/stan/agrad/fwd/matrix/qr\_R.hpp Source File


|  |  |
| --- | --- |
|  | Stan  2.5.0  probability, sampling & optimization |


- Main Page
- Related Pages
- Namespaces
- Classes
- Files

- File List
- File Members

All Classes Namespaces Files Functions Variables Typedefs Enumerations Enumerator Friends Macros Pages

- src
- stan
- agrad
- fwd
- matrix

qr\_R.hpp

Go to the documentation of this file.

1 #ifndef STAN\_\_AGRAD\_\_FWD\_\_MATRIX\_\_QR\_R\_HPP

2 #define STAN\_\_AGRAD\_\_FWD\_\_MATRIX\_\_QR\_R\_HPP

3

4 #include <stan/math/matrix/Eigen.hpp>

5 #include <Eigen/QR>

6 #include <stan/math/error\_handling/matrix/check\_nonzero\_size.hpp>

7 #include <stan/math/error\_handling/check\_greater\_or\_equal.hpp>

8 #include <stan/agrad/fwd/fvar.hpp>

9

10 namespace stan {

11  namespace agrad {

12

13  template <typename T>

14  Eigen::Matrix<fvar<T>,Eigen::Dynamic,Eigen::Dynamic>

15  qr\_R(const Eigen::Matrix<fvar<T>,Eigen::Dynamic,Eigen::Dynamic>& m) {

16  typedef Eigen::Matrix<fvar<T>,Eigen::Dynamic,Eigen::Dynamic> matrix\_fwd\_t;

17  stan::math::check\_nonzero\_size("qr\_R(%1%)",m,"m",(double\*)0);

18  stan::math::check\_greater\_or\_equal("qr\_R(%1%)",m.rows(),m.cols(),"m.rows()",

19  (double\*)0);

20  Eigen::HouseholderQR< matrix\_fwd\_t > qr(m.rows(), m.cols());

21  qr.compute(m);

22  matrix\_fwd\_t R = qr.matrixQR().topLeftCorner(m.rows(),m.cols());

23  for (int i=0; i<R.rows(); i++) {

24  for (int j=0; j<i; j++)

25  R(i,j) = 0.0;

26  if (i < R.cols() && R(i,i) < 0.0)

27  R.row(i) \*= -1.0;

28  }

29  return R;

30  }

31  }

32 }

33 #endif

stan::agrad::fvar

**Definition:** fvar.hpp:13

stan::agrad::qr\_R

Eigen::Matrix< fvar< T >, Eigen::Dynamic, Eigen::Dynamic > qr\_R(const Eigen::Matrix< fvar< T >, Eigen::Dynamic, Eigen::Dynamic > &m)

**Definition:** qr\_R.hpp:15

fvar.hpp

Eigen.hpp

check\_greater\_or\_equal.hpp

check\_nonzero\_size.hpp

stan::math::check\_greater\_or\_equal

bool check\_greater\_or\_equal(const char \*function, const T\_y &y, const T\_low &low, const char \*name, T\_result \*result)

**Definition:** check\_greater\_or\_equal.hpp:58

stan::math::check\_nonzero\_size

bool check\_nonzero\_size(const char \*function, const T\_y &y, const char \*name, T\_result \*result)

Return true if the specified matrix/vector is of non-zero size.

**Definition:** check\_nonzero\_size.hpp:33


---

[ Stan Home Page ]

*© 2011–2014,
Stan Development Team.*
